# From Mind to Milk: The Influence of Psychological Factors on the Composition of Human Breast Milk

**DOI:** 10.3390/nu17061093

**Published:** 2025-03-20

**Authors:** Krystian Skowron, Igor Lichocki, Filip Godziszewski, Magdalena Orczyk-Pawiłowicz

**Affiliations:** 1 Student Scientific Association of Medical Chemistry and Immunochemistry, Wroclaw Medical University, 50-367 Wroclaw, Poland; 2 Student Scientific Club of Drug Form Technology, Wroclaw Medical University, 50-367 Wroclaw, Poland; igor.lichocki@student.umw.edu.pl; 3 Clinical and Dissecting Anatomy Students’ Scientific Club, Wroclaw Medical University, 50-367 Wroclaw, Poland; filip.godziszewski@student.umw.edu.pl; 4 Division of Chemistry and Immunochemistry, Department of Biochemistry and Immunochemistry, Wroclaw Medical University, M. Skłodowskiej-Curie 48/50, 50-369 Wroclaw, Poland

**Keywords:** breastmilk composition, lactation, maternal stress, postpartum depression, psychological factors, nutrients, microbiota, hormones, immune factors

## Abstract

**Background/Objectives:** Breast milk is a complex fluid crucial for infant development, nutrition, and immunological and neurodevelopmental support. Recent findings suggest that factors regarding mental health, such as stress, anxiety, and postpartum depression (PPD), may influence the composition of breast milk. This review aims to synthesize current knowledge regarding the relationship between a mother’s mental state and the biochemical profile of human milk, focusing mainly on nutrients, hormones, immune factors, and microbiota. **Methods**: A systematic literature search was conducted in PubMed and the Web of Science using predefined keywords related to psychological factors and milk composition. Studies involving validated psychological assessment tools and only human subjects were included, in accordance with PRISMA guidelines. **Results**: Findings indicated that maternal stress and PPD are associated with alterations in breast milk composition. Elevated cortisol and changes in melatonin and prolactin levels have been observed. Immune components, such as secretory immunoglobulin A and transforming growth factor beta 2, exhibit variable responses depending on stress type and duration. Lower concentrations of docosahexaenoic acid and polyunsaturated fatty acid have been observed among mothers diagnosed with depression. Additionally, maternal psychological distress may influence infants’ gut microbiota composition, potentially affecting long-term health outcomes. **Conclusions**: The maternal psychological state plays an essential role in shaping the composition of human breast milk. Understanding these associations highlights the need for mental health support during the postpartum period to optimize infant development. Future research should focus on the molecular mechanisms underlying these changes and potential interventions to mitigate adverse effects.

## 1. Introduction

Breast milk, secreted by lactocytes and pink adipocytes, is a heterogeneous solution containing a wide range of compounds that not only nourish the newborn, but also shape development [1,2,3,4]. It is recommended to breastfeed exclusively for at least the first six months of an infant’s life and continue breastfeeding with additional nutrition until the infant reaches their second year [5,6]. During child growth and development, the content of milk must change to fulfill actual needs [2,7]. Human breast milk is divided into three categories, based on lactational age, that corresponds with its composition [8]. In the beginning, colostrum is produced until 5 days postpartum. Compared to the milk in further stages, it has lower lactose, fatty acid, calcium and potassium levels, but is enriched in sodium, chlorides, and magnesium [9]. The main role of colostrum, apart from nutrition, is to participate in newborns’ adaptation to the extrauterine environment, in which it is imperiled by surrounding microorganisms. This protective function is manifested by high levels of immunoactive factors [2,10,11]. From 6 to 15 days postpartum, transitional milk is secreted. During this period, junctional closure in mammary epithelium tightens, which results in a decline in sodium–potassium ratio and an increased lactose content [2,12]. Mature milk emerges after 15 days postpartum and serves as the main source of nutrients for the infant. Immune and growth factors decrease in favor of micro- and macronutrients [2,13,14].

Apart from changes in the composition associated with the lactation stage, evidence shows that maternal mental state may shape the content of many compounds in breast milk [15]. Elevated stress levels during pregnancy, labor, and early stages of parenthood are reasonable. In fact, some scales, like The Social Readjustment Rating Scale, are used to measure stress level and attribute high value to this type of issue [16]. A problem arises when anxiety or a mood disorder becomes pathological. Current data indicate that around 85% of women deal with emotional disturbance while in postpartum period [17], which shows how serious the issue of a maternal psychological condition is [18].

Postpartum depression (PPD) is one of the most serious mental conditions and it affects around 10–15% of mothers [18,19,20]. The connection between PPD and breastfeeding is rather complex, with some papers showing a protective effect [21] and others lean towards considering it as a PPD risk factor. Alterations in maternal metabolomes during PPD are confirmed [22], leading to a lot of research in which connections between mental state and changes in milk composition are investigated. The biological model of PPD underlines the role of chronic low-grade inflammation, hormonal imbalance, and disturbance in neuro-active species like kynurenine [23,24]. Similar biological changes have been observed in a state of perinatal anxiety or stress [25]. Inflammation also impacts mammary gland epithelium permeability, thus indirectly changing the milk’s composition [26]. Another indirect change in breast milk content may be attributed to a deleterious habit, for example, substance abuse or improper diet, which occur more frequently in an altered mental state, such as PPD [27].

Components of human breast milk also affect metabolic and neurobiological development which may impact infant’s whole future life in terms of physical and mental well-being [28,29,30,31,32,33]. It is called “lactation programming hypothesis” [28]. Metabolic dysregulation in newborns is also attributed to the increased risk of mental health conditions, including depression in adolescent life. This association highlights the importance of early-life experiences in shaping long-term psychological outcomes [34]. Interestingly, breastfeeding has been identified as a protective factor, with studies showing that it may reduce the risk of depression in later life by modulating metabolic and hormonal pathways that influence brain development [35,36]. Additionally, research shows that breastfeeding may have a protective influence in developing autism spectrum disorder. The mechanism is unclear; however, it is probably due to the contents of colostrum, which mediate neuro- and sociodevelopment [37]. Apart from skin-to-skin contact, oxytocin is an important factor facilitating the behavioral maturation of an infant. Oxytocin is described as compound promoting social behavior while preventing aggression and antisocial demeanor [38]. The association mentioned is an example of how the mother’s mental state may shape offspring’s psychological condition via breast milk. Apart from the direct effects of hormones or growth factors on a child, breastfeeding also impacts the infant’s gut–brain axis, a complex communication network between the gastrointestinal tract and the brain [35,39]. Abnormally high gut permeability is a known factor link with many conditions such as multiple sclerosis, Parkinson’s disease, allergies, or mentioning depression and the autistic spectrum [40,41,42,43]. A recent study showed that the main factors influencing infant’s gut microbiota composition are vitamin D levels in cord blood, race, delivery method, and breastfeeding [44]. The gut–brain axis also modulates various processes, the most known being the immune response, but it is established that bacterial metabolites take part in epigenetic changes, thus potentially modulating a vast number of systems and responses [39,45].

**Figure 1:**
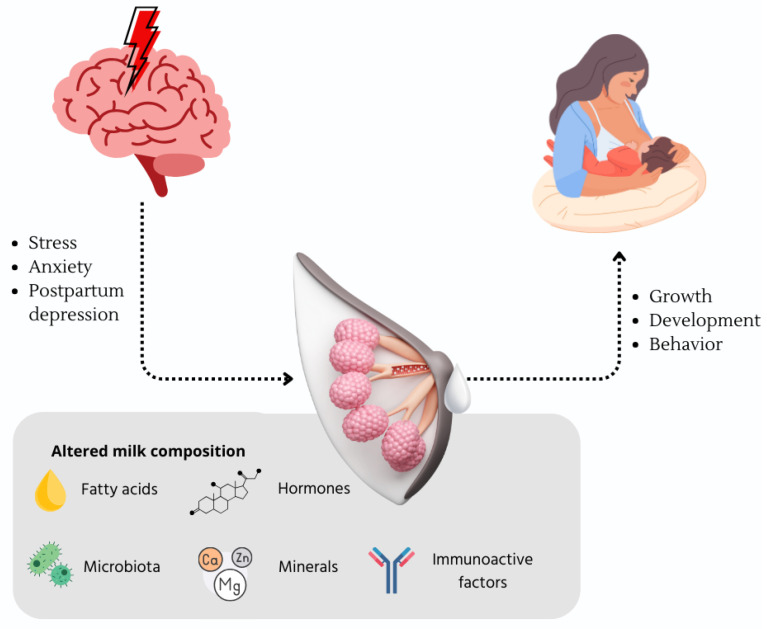
The relationship between maternal mental state and offspring’s development is mediated via breast milk.

The aim of this review was to summarize current knowledge on the connection between psychological factors, measured by a well-known, established questionnaire, and levels of compounds detected in human breast milk (Figure 1). Only one article was published on a similar topic, but plenty of research was conducted after its publication; hence, there is need for an updated review [46].

## 2. Materials and Methods

**Figure 2:**
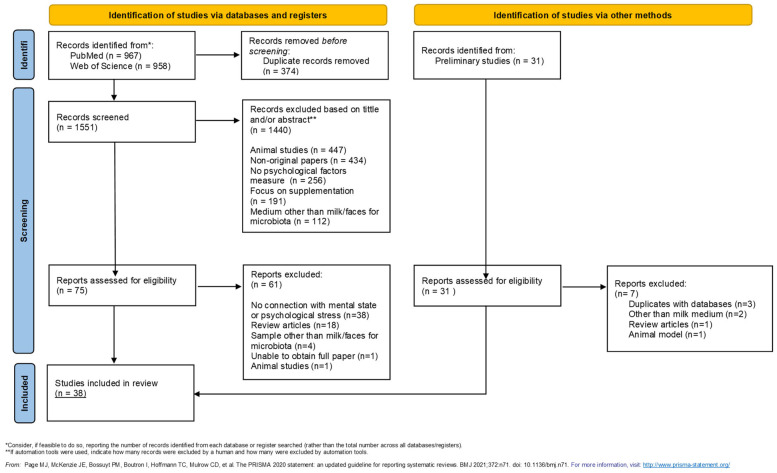
PRISMA flowchart [47].

Preliminary studies have been conducted to specify the topic of the paper and identify possible factors and components that should be included in the database search. We conducted a systematic search in PubMed and Web of Science using a three-part query with proper Boolean terms (AND to link words in proper part, OR to link all 3 parts): (1) specifying species—only human; (2) impaling varieties of psychological factors; (3) compounds found and studied in human breast milk. The best result was achieved while using in part 2 and 3 words: postpartum depression, depression, stress and trauma in combination with lactating women, breastfeeding, lactation, human milk, breast milk, composition, acid, fatty acid, gastrointestinal flora, microbiota, lactic acid bacteria, Lactobacillus, microbiome, immunoactive factors, transforming growth factor beta, antibodies, immunoglobulins, IgA, retinoids, sodium, potassium, and ions. A language filter, set for English, was imposed on both bases and no publication date restrictions were imposed. The course of paper selection with all criteria is shown in the PRISMA chart (Figure 2). Manually found preliminary studies that fit the topic were also included. As shown in the chart, this source consists of 31 papers. The review contains 38 papers published up to 5 December 2024. Two independent reviewers participated in data processing by using the PubMed and Web of Science databases. Reviewers screened 1551 records and, based solely on the title and abstract, only 75 were chosen for further assessment. Most original papers were excluded due to conducting studies on animals, not involving psychological factors, or focusing on supplementation. Given the constraints of the topic, microbiota research relied on fecal samples. These studies are nevertheless included, as they provide indirect evidence of the relationship between breast milk and the development of gut microbiomes.

## 3. Results

The findings were divided into predefined categories; some papers were shown in more than one category due to the investigation of multiple compounds (Table 1). Fatty acids were excluded from the category “general nutrients” due to the abundance of articles that they were the subject of and are discussed in a separate subcategory. Due to the nature of the subject, microbiota studies had to be conducted on fecal samples but were included as they indirectly illustrate the association between breast milk and the gut microbiome shaped by this important fluid.

**Figure 3:**
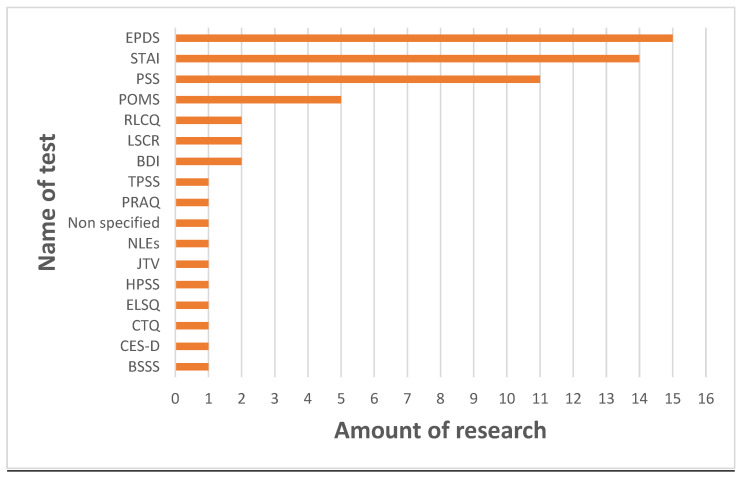
Frequency of usage of individual tests (one publication may be included in more than one category). **Abbreviations:** BDI—Beck Depression Inventory; BSSS—Berlin Social Support Scale; CES-D—Center for Epidemiologic Studies Depression Scale; CTQ—Childhood Trauma Questionnaire; ELSQ—Early Life Stress Questionnaire; EPDS—Edinburgh Postpartum Depression Scale; HPPS—Hung Postpartum Stress Scale; JTV—Jeugd Trauma Vragenlijst; LSCR—Life Stressor Checklist—Revised; POMS—Profile of Mood States; PRAQ—Pregnancy-Related Anxiety Questionnaire—Revised; PSS—Perceived Stress Scale; RLCQ—Recent Life Changes Questionnaire; STAI—State-Trait Anxiety Inventory; TPSS—Tennessee Postpartum Stress Scale.

**Figure 4:**
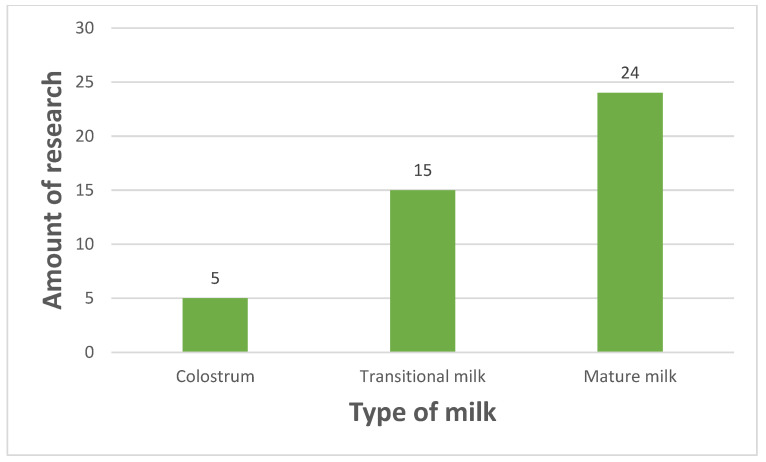
Amount of research conducted on milk type (one publication may be included in more than one category).

Categories that contain most articles are hormones and immunological factors (both include 14 findings). It is reasonable due to the nature of the subject that implies searching for changes in stress-associated compounds (this trait is attributed to hormones, mainly cortisol) and compounds that have a noticeable role (most people connect breast milk mostly with immunological properties). Most commonly, the Edinburgh Postpartum Depression Scale (EPDS) and State-Trait Anxiety Inventory (STAI) test were used. Of the studies included, 39% used EPDS and 37% used STAI. The use of the latter is especially noticeable when considering traits and state anxiety, hence giving more information about characteristics of stress. Some tests were only applied in one study (3% of included studies). These tests are the Berlin Social Support Scale; the Life Stressor Checklist—Revised; the Tennessee Postpartum Stress Scale; the Early Life Stress Questionnaire; the Childhood Trauma Questionnaire; the Life Stressor Checklist—Revised; Jeugd Trauma Vragenlijst, the Center for Epidemiologic Studies Depression Scale; and the Pregnancy-Related Anxiety Questionnaire—Revised. The prevalence of test usage is illustrated in Figure 3. Most research was conducted on mature milk (24 of 38 included studies), approximately half of studies examined transitional milk (15 of 38), while only 5 of 38 investigated changes in colostrum (Figure 4); hence, mainly long-term stress was assessed.

### 3.1. General Nutrients

This category included all papers investigating general nutrition values alongside amino acid concentration (Table 1). Only two articles measured the whole composition of mothers’ milk. To sum up, the results of both studies give an insight into all three types of milk, including total calorie content and macronutrients such as protein, fat, and carbohydrates [49,51]. Unambiguous results were presented in these papers, supporting the thesis that postpartum depression or stressful life events do not significantly change fat and energy contents of human milk (HM). Carbohydrates and fat contents were also included in another study, which focused more on cortisol levels in mature milk. Nevertheless, they did find a negative correlation between recent life changes (RLCs) and fat content, while RLCs were not correlated with carbohydrates [48]. Lastly, we found one article each about the oligosaccharide composition in mature milk [52] and amino acid concentration in both transitional and mature milk [50]. In a paper researching amino acids, protein-bound amino acids’ (BAAs) levels were higher in mothers’ milk in the high-stress group compared to the control group. Also, free methionine was higher in the high-stress group, but in the end only BAAs were positively associated with high stress levels one month postpartum. Methionine correlation was not statistically significant [50]. Human milk oligosaccharides (HMOs) were investigated in the final article. The results showed that a couple of HMOs varied with maternal psychological state, and it was hinted that there could be a possible relation between HMOs and maternal stress and depression; however, further studies need to be conducted to confirm this relationship [52].

New findings revealed that the intake of common drugs, such as selective serotonin reuptake inhibitors (SSRI), anti-inflammatory drug, or steroids, significantly impacts milk composition in terms of general nutritional value. The concentrations of protein, fatty acids, and energy content differed significantly in the population of mothers who were treated with the drugs mentioned, while carbohydrate levels remained constant. From the perspective of this review, the presence of SSRI drugs that are commonly used to treat mood disorders like PPD is especially important. It is important to state that this finding does not change any recommendation regarding the use of those drugs during lactation [87].

### 3.2. Fatty Acids

We gathered four studies about fatty acids associated with postpartum depression. Three out of the four studies investigated the levels of DHA content in mothers’ mature milk [53,54,56], whereas one study examined fatty acids like SFA, monounsaturated fatty acid (MUFA), or PUFA in transitional and mature milk [55]. The results of one study focused on seafood consumption and DHA levels showed that maternal postpartum depression is associated with lower concentrations of fatty acids in HM. This was a cross-national analysis and, after excluding several extreme values, these regression models showed heavy associations between high DHA levels in mothers’ milk and higher sea food consumption with a lower risk of postpartum depression [53].

### 3.3. Microbiota

Gut microbiota is very important in terms of postnatal development [57]. Only 3 papers about gut microbiota were included in our study [57,58,59]. Methods of quantification differed in 2 studies [57,58], where the stool sample was collected to accurately determine gut composition. Also, in these studies, an evaluation of participants’ mental state was conducted for the prenatal period. Different conclusions were drawn from selected papers. A high count of bacteria from group PRO1 and low number of bacteria from groups LAB and ACT1 were strongly associated with prenatal cumulative stress, which led to the conclusion that maternal stress can drastically change infant microbiota [58]. Another study found that there was a trend towards a higher variety of bacteria being present in mothers’ milk with less maternal stress; however, at the third and seventh day postpartum, where mothers experienced higher stress levels, the diversity of microbiota was increased. This phenomenon was explained by intestinal dysbiosis [59]. A similar conclusion was made in another study, in which prenatal depression was associated with notable changes in the microbiota diversity of non-breastfed infants. However, no changes were caused in microbiota diversity in fully breastfed infants by maternal prenatal mood status [57].

The findings presented align with reports linking mental state and microbiota. Stress-related dysbiosis is not only characterized by altered bacterial diversity but also by significant shifts in the metabolic capabilities of the gut microbiota, potentially affecting neurodevelopment through modifications in microbial metabolite profiles. This connection opens new possibilities in the application of probiotics to psychiatric treatment [88,89].

### 3.4. Immunological Factors

This category is very heterogeneous, but most findings involve determining the connection between maternal stress and the level of secretory immunoglobulin A (SIgA) (Table 1). Out of 14 included studies, 12 measured levels of SIgA in all three kinds of breast milk [61,63,64,66,67,68,69,70,71,72,73], and two of these studies also measured other immunoglobulins in mature milk: IgM and IgG (both papers show no correlation) [67,68]. The results of SIgA studies are ambiguous. A relationship was established between daily mood and SIgA in saliva [90], showing that a similar mechanism may be involved in secretory immunoglobulin production. A wide range of different outcomes may be contributed to examining different milk species (colostrum, transitional or mature) and perceiving stress in different ways, which means evaluating by different scales. Data show that SIgA levels are connected to acute stress rather than chronic stress [91].

Two growth factors, TGFβ2 and EGF, were also investigated. Only one study measured EGF levels and showed reduced concentrations in colostrum and transition milk [69]. Articles about TGFβ2 report a negative correlation, no correlation, and positive correlation [62,63,65,66]. Investigations were mainly conducted on mature milk; one study additionally tested transitional milk. One of the studies reporting a negative correlation mentioned that the use of more advanced statistical methods does not demonstrate a change in the concentration due to the maternal psychological state (human milk cortisol and immune factors over the first three postnatal periods) [67]. TGFβ2 is known for participating in immunity acquisition. In colostrum, it may serve as a starter for IgA production; thus, the mutual connection between the two is also worth noting [92]. Additionally, recent studies show that TGFβ2 exhibits a role in lung development and fibrosis [93]. In depressed populations, no correlations were observed between mean serum TGFβ2 levels and mental state [94]. However, considering HM, it is also important to take into account local inflammation that makes mammary glands more permeable, thus increasing TGFβ2 in HM [95,96]. Apart from these compounds, one study showed a negative correlation between the STAI score and lactoferrin concentration in mature milk [68]. Two studies measured multiple immunoactive compounds in all three types of milk (mainly cytokines such as IL-4, IL-6, IL-8, IL-10, MCP-1, IP-10, TNFα), but no correlation was observed [67,69].

### 3.5. Hormones

Cortisol levels were quantified. This is understandable considering the presence of cortisol in the stress response reaction [97]. We found ten papers that investigated the link between breast milk’s cortisol levels (in all stages of milk maturation) and the maternal psychological state (Table 1). Only one article found lowered cortisol levels [71], but only in transitional milk, whereas other studies conducted at similar times, between 6 and 15 days postpartum, showed either elevation or no correlation in cortisol levels [60,67,73,78,80]. Studies that involve milk several months postpartum conclude that there is no connection between cortisol in milk and the results of tests assessing maternal mental state [74,76,77,81]. Cortisol, when maintained within specific physiological ranges, supports the regulation of infants’ emotional, behavioral, and autonomic functions. However, in cases of certain congenital disorders, there is an association with cognitive deficits and disruptions in the infant’s autonomic nervous system [98,99]. Chronic stress impacts the hypothalamic–pituitary–adrenocortical (HPA) axis activity in a dynamic and context-dependent manner. The findings suggest that HPA activation is heightened at the onset of stress but diminishes over time. Moreover, individual responses play a critical role, with subjective distress amplifying HPA activity, while some conditions are associated with reduced activity. These results underscore the complex interplay between stress characteristics, individual differences, and HPA function, hence explain the hardships in establishing connections between cortisol levels and stress when these stress are simply described by a questionary score [100,101].

One study showed that stress alters maternal mature milk by increasing melatonin and decreasing prolactin [75], but most recent studies have shown no correlation between stress and prolactin levels (no further studies investigating melatonin were found) [72]. The results are interesting since PPD, like other depression types, is linked to elevated oxidation stress. Melatonin was proven to be an antioxidant; thus, elevated levels may be connected to higher antioxidants demands [102,103]. Apart from this, it was proven that melatonin impacts neurogenesis [104]. Prolactin results are equally interesting because recent studies have shown that females with major depression exhibit high levels of serum prolactin [105]. Milk-derived prolactin contributes to the functional maturation of the neuroendocrine and immune systems; hence, the low levels exhibited during stress may impair these processes [106]. This finding may be crucial in explaining the heritability of depression and key factors predisposing people to mental disorders.

A study that examined the steroid hormone profile of human mature milk showed elevated pregnenolone, a substrate for further steroid synthesis; however, no other connection was found [77]. Additionally, one study found a positive correlation in colostral β-endorphin, which was attributed to maternal anxiety levels [79]. Both cortisol and β-endorphin have a common origin in proopiomelanocortin, which may suggest that changes in their levels should be correlated. Endorphin plays a similar role in child development to other neurohormones found in HM. It modulates the immune system by suppressing the proinflammatory cytokines and stimulating anti-inflammatory cytokines; hence, it may be a mechanism compensating the elevated proinflammatory cytokines produced in depressive episodes or elevated stress situations [17,107]. Evidence also shows that umbilical cord β-endorphin exhibits a role in motor development of a child [108].

### 3.6. Ions

Selected studies examined the different ions present in HM, mainly Na^+^ and K^+^ and their ratios, but one study also examined trace elements like selenium and iron (Table 1). All studies were conducted on transitional or mature milk. Mixed conclusions were reached in studies about ions present in mothers’ milk. Two studies suggested that there is no correlation between ions level in HM and maternal stress [82,85]. In the case of the first study, increased mammary gland permeability was slightly correlated with postpartum depression. The Na/K ration was higher in 29% of women admitted to this study. Its correlation with postpartum depression was marginally significant; however, this paper only featured small and non-randomly selected breastfeeding women. A trace elements study came to the similar conclusion that postpartum depression does not change ions level in HM [82]. This is the same conclusion that was reached in another study, where the Na^+^/K^+^ ratio was examined [71]. On the other hand, different studies showed that maternal stress and psychosocial characteristics can influence ion level. Mothers with higher Na^+^ levels had notably higher STAI scores than mothers with low Na^+^ levels, and mean EPDS scores were also significantly higher in mothers with high Na^+^ concentrations and high Na^+^/K^+^ ratios [84]. Results of the last study suggest that mothers with higher breast milk sodium levels and hypernatremic dehydration tend to have a history of psychiatric morbidity [83].

### 3.7. microRNA

Only one study was found about microRNA residing in mature milk [86]. It is suggested that RNA encapsulated in extracellular vesicles (EVs) is responsible for gene regulation and signaling pathways between the mother and the infant during early pregnancy. Breast milk was collected from mothers around 6 weeks postpartum; then, EVs were isolated. Negative correlations were found between the number of isolated EVs-microRNA and LSCR scores. After adjusting for infant sex, maternal race, education, and postpartum week of breast milk collection, 60 microRNAs (35%) were associated with higher LSCR scores. Scientists also noticed a trend towards less EV-microRNAs with higher a Negative Life Event (NLE) score during pregnancy period; however, it was not statistically significant. Overall, stress, not only during pregnancy, was positively associated with the EV-microRNA expression count [86].

Recent research highlights extracellular RNA communication as a crucial mechanism of intercellular signaling, particularly during pregnancy, where it plays a role in embryo implantation, remodeling of the uterine spiral artery, immune modulation, and responses to inflammation. Evidence emerging from plasma-based studies further emphasizes the role of EV-mRNA in postpartum mental health. These studies analyze EV-mRNA profiles in maternal blood at multiple time points throughout pregnancy and postpartum. Substantial alterations have been reported in women who later developed PPD [109]. These findings open the possibility of utilizing EV-mRNA as a biomarker for the early detection of PPD and stress-related disorders, as well as a potential target for therapeutic purposes.

Given that EVs in human milk originate from maternal circulation and play a pivotal role in neonatal development, it is plausible that the molecular changes observed in plasma EV-mRNA may also be reflected in milk-derived EVs. Future research should explore whether alterations in EV-mRNA content in human milk correlate with maternal stress, depression, and immune dysregulation [109].

## 4. Discussion

In recent years, there have been several publications evaluating changes in the composition of human breast milk and articles involving the maternal psychological state. We presented the largest up-to-date review, focusing on the changes in specific compound groups, which allowed us to see a broader perspective on the role of breast milk in shaping future life. This review was enriched in the section describing microbiota, and it is a trend to investigate the connections between humans and their microbiomes. Unfortunately, only a few studies were used in our paper; however, we can see a trend that in women with stress and anxiety, there are some changes in microbiota. Lower stress levels are associated with more significant microbiota diversity. Lactic acid bacteria and the *Actinobacter* group were the most susceptible to maternal stress, but responses differ in cases of low and prenatal stress.

While numerous studies have explored the link between the maternal psychological state and breast milk composition, the complexity of stress as a phenomenon presents challenges in establishing clear connections. To the best of our knowledge, only one review exists on a similar topic. While authors of the previous article focus on the impact that altered milk has on child neurodevelopment, we chose to present updated results in a more systematic manner that facilitates the drawing of conclusions [46]. This review also underlines the role of experiment preparation manifested in choosing the time of sampling, as well as the method of chemical analysis allowing critical data assessment. It is also the first review to include microRNA and microbiota, which are trending subjects in current biomedical research. Maternal psychological state is measured using many scales (Figure 3); therefore, it is important to underline which aspect of the psychological condition is being evaluated. Some conclusions were drawn with short-term stress, while other studies focused on long-term effects that were closer to PPD. Some tools assess the maternal state in a more holistic way by using mood scales rather than simple numerological values.

As mentioned in this review, the connection between child neurodevelopment—and even traits, such as temperament—and HM composition was established [29,75,110]; thus, milk should be considered as a factor that shapes the whole future life of a persons’ offspring. That means more attention should be paid to providing mothers with appropriate psychological help if needed, especially during times of early maternity and breastfeeding. There are still fields that lack evidence, and some compounds were only examined in one or two studies. Considering the connection between depression and oxidative stress, it is unusual that no papers measuring oxidative stress markers were found [103]. Additionally, even though vitamin D was found in human breast milk8, there is no article that investigates the connections between the mentioned vitamin and maternal psychological state. Reasons that advocate for vitamin D quantification lay in the role of these molecules [111]. For breastfeeding mothers, vitamin D is important for maintaining calcium homeostasis, hence ensuring proper calcium level in milk. Additionally, antioxidative properties are attributed to this vitamin, which is especially important in oxidative stress present in pathologies such as PPD. Recent studies also marked vitamin D as a promising indicator for PPD because of the correlation between antenatal blood vitamin D levels and PPD symptoms [112,113].

Future research examining the connection between psychological factors and the composition of human breast milk could investigate molecular mechanisms that will explain the changes presented in this review. Better understanding may benefit in identifying specific biomarkers to monitor maternal mental health, which will provide psychiatric biomedical tools that this branch of medicine generally lacks during diagnosis. Another course of study may involve the impact that changed milk has on infants’ development and how the supplementation of specific compounds may prevent those negative changes. Connection with neurodevelopment is well established, but as was stated in this article, compounds of human breast milk impact many different processes. Thus, more research is needed [46,114,115].

Approximately 70% of lactating mothers use medications during breastfeeding. There is research about the safety of drug usage, but we still lack investigations into how the composition of breastmilk is being changed due to the use of this medication. The impact of selective serotonin reuptake inhibitors on macronutrients was described just recently, but more research is still needed to gain an insight into how SSRI changes other components important for breastfed babies [88]. Other psychiatric medications may also need to be verified.

From the perspective of public health, research findings in these areas could have significant implications for healthcare providers and policies aimed at improving maternal and child health. Understanding the impact of psychological well-being on breastfeeding could lead to more targeted interventions that address both the mental health needs of mothers and the nutritional needs of infants [116]. Integrating mental health support into prenatal and postnatal care programs could enhance the overall quality of breastfeeding, thereby reducing the risk of poor infant health outcomes associated with inadequate milk composition. Additionally, such insights could inform public health campaigns that promote not only breastfeeding but also maternal mental health, ultimately contributing to healthier populations.

## 5. Conclusions

This review underlines the substantial influence of maternal psychological factors, including stress, anxiety, and postpartum depression, on the composition of human breast milk. Alterations in key elements such as immunological factors, hormones, fatty acids, microbiota, and nutritional value highlight the intricate link between breast milk and maternal mental health. The long-term implications for various aspects of infant health, personality, and development remain an area of ongoing research.

Despite advancements, inconsistencies in study methodologies (particularly in the quantification of psychological factors) and limited comprehension of the underlying mechanisms present challenges in drawing definitive conclusions from a multitude of results (Figure 1). Further investigation is warranted to explore the molecular pathways, identify biomarkers, and assess the specific impacts of altered milk composition on infant health. Such efforts would yield substantial benefits for both mothers and their children.

Hormonal alterations, such as elevated cortisol and pregnenolone levels, were reported in most of available papers, while some studies indicated changes in prolactin and melatonin concentrations. Immunological factors, particularly secretory immunoglobulin A, displayed variable responses, with acute stress often linked to increased levels and chronic stress linked to reductions. Fatty acid profiles showed decreases in DHA and other polyunsaturated fatty acids (PUFAs) in mothers experiencing psychological distress. Changes in nutritional value, such as amino acid composition, were also observed, with higher protein-bound amino acids seen in stressed mothers. Additionally, maternal mental health influenced the diversity and composition of infant gut microbiota, particularly when stress was experienced during pregnancy.

From a public health perspective, maternal mental health is a serious issue and the impact that psychological factors play in breastfeeding make it even more significant. Addressing maternal well-being not only benefits mothers but also ensures optimal breastfeeding practice, thereby fostering improved health outcomes for future generations. Such an approach aligns with the current doctrine of holistic patient care.

## Figures and Tables

**Table 1:** Impact of psychological factors on human breast milk composition.

Ref.	Year	Sample Size	Method of Evaluating Mental State	Time of Evaluating Mental State (Days/Weeks/Months After Giving Birth)	Method of Analysis	Time of Milk Sampling (Days/Weeks/Months After Giving Birth)	Results	Additional Information
General nutritional value (n = 6)								
[48]	2021	146	RLCQ	5 months	MID-IR	5 months	↓ energy density	
[49]	2023	75	EPDS	4 weeks	MID-IR	no longer than 6 months	No correlation: total protein and carbohydrates concentration, calorie content	
[50]	2023	116	LSCR + EPDS + STAI + JTV	10 days	LC-MS/MS	10, 17, 24 days	↑ bound AAs, ↑ Free methionine	BAAs positively associated with cortisol No correlation found for bound methionine
							No correlation: free AAs	
[51]	2020	21	STAI-T + STAI-S	STAI-T + STAI-S—1 day after admission	IR transmission spectroscopy	1 and 2 days after admission and 1 week after discharge	Energy content ↑ on day 7 compared with admission	No significant differences in fat and energy content between days (1 to 2 and 2 to 7)
				STAI-S—1 week after discharge				
[52]	2024	926	EPDS, STAI, PSS	60 days	HPLC	60 days	Change in oligosaccharides: lacto-N-fucopentaose III, lacto-N-hexaose, disialyl-lacto-N-hexaose	
Fatty acids (n = 6)								
[49]	2023	75	EPDS	4 weeks	MID-IR	no longer than 6 months	No correlation: total FA content	
[51]	2020	21	STAI-T + STAI-S	STAI-T + STAI-S—1 day after admission	IR transmission spectroscopy	1, 2 days after admission and 1 week after discharge	FA ↑ on day 7 compared with admission	No significant differences in fat and energy content between days (1 to 2 and 2 to 7)
				STAI-S—1 week after discharge				
[53]	2002	14 532	EPDS	4–240 days	varies depending on source *	varies depending on source *	↓ DHA	AA and EPA content in HM were unrelated to PPD prevalence
[54]	2023	115	EPDS	30–120 days	GC-MS	30–120 days	↓ DHA	
[55]	2022	116	PSS, EPDS, STAI	10 days	GC-FID	10, 17 and 24 days	↓ FA, PUFA, ω-6 PUFA	Changes observed only in mature milk and not in transitional
[56]	2012	287	CES-D Scale	before 20 weeks and at 24–29 weeks of pregnancy	chromatography with standards comparison	4 months	↓ DHA	
							No correlation: other than DHA FA	
Microbiota ** (n = 3)								
[57]	2021	996	Non-specified	prenatal	QIAamp DNA Stool Mini Kit	3–4 months	changes in microbiota in infants who are not exclusively breastfed	Microbiota composition was not affected by mood in group infants breastfed at 3–4 months
[58]	2015	192	STAI + PRAQ	prenatal (35 week)	Human Intestinal Tract Chip	7, 13, 25, 84, 112 days	low stress = ↑ LAB and ACT1	
							prenatal stress = ↑ PRO1, ↓ LAB, ACT1	
[59]	2023	52 newborns and 45 mothers	parental stress scale PSS:NICU	3, 7 and 15 days	MilkoSkanTM Mars, FOSS, Hilleroed, Denmark	3, 7 and 15 days	↑ microbiota diversity = ↓ stress	Results were not statistically significant
Immunological factors (n = 14)								
[60]	1994	34 preterm + 29 terms	POMS	5 days	RID	5 days	Preterm: ↑ SIgA (anger)	There is no significant correlation between value and anxiety score
							Term: no correlation: SIgA	
[61]	2004	50	PSS, POMS	10 weeks	ELISA	10 weeks	↑ SIgA	
[62]	2011	139	EPDS	3 months	ELISA	3 months	↑ TGFβ2	
[63]	2014	84	POMS	6 weeks	ELISA	2–4 days and 6 weeks	↑ SIgA	Significant, positive correlation between colostrum SIgA and anger (subcategory of mood state) was reported
							No correlation: TGFβ2	
[64]	2015	81	POMS	2 weeks	ELISA	2 weeks	↓ SIgA	
[65]	2017	110	STAI, BDI	4–6 months	ELISA	4–6 months PP	↑ TGFβ2	
[66]	2019	89	Hung Postpartum Stress Scale +PSS	4–6 weeks	ELISA	4–6 weeks	↓ SIgA	
[67]	2020	51	STAI-S, EPDS	6 weeks	ELISA	2, 6, 12 weeks	↑ IL-8 * ↑ IL-7 * ↓ TGFβ2	
							No correlation: IgA, IgG, IgM, IL-1, TNFα, MCP1, MIP1β, GROα, IL-5, EGF,	
[68]	2021	103	STAI + BSSS	5 months	ELISA	>5 months	↓ Lactoferrin ↓ SIgA	
							No correlation: IgG, IgM	
[69]	2016	85	PSS	3 and 9 days	ELISA (SIgA), Luminex	3, 9, 14 days	↓ EGF ↓ MIP1α ↓ TNFα	
							No correlation: SIgA, IL-4, IL-6, IL-8, IL-10, MCP-1, IP-10	
				14 days			↑ IL-8 ↑ MIP1α	
							No correlation: SIgA, EGF, IL-4, IL-6, IL-10, MCP-1, IP-10, TNFα	
[70]	2022	2310	PSS, LSCR	0–12 months	ELISA	up to 12 months	↓ SIgA (SARS-CoV-2-specific antibodies)	
[71]	2024	26	EPDS, STAI	5 weeks	Immunoassay	1 and 5 weeks	↑ SIgA (only week 5)	
[72]	2004	55	STAI	1–8 months	ELISA	1–8 months ^	No correlation: SIgA	
[73]	2004	32	BDI, POMS	7–11 days	Nephelometry	7–11 days	↑ SIgA	
Hormones (n = 13)								
[60]	1994	34 preterm + 29 terms	POMS	5 days	RID	5 days	Preterm: no correlation: cortisol	
							Term: no correlation: cortisol	
[67]	2020	51	STAI-S, EPDS	6 weeks	LC-MS	2, 6, 12 weeks	↑ Cortisol	
[74]	2024	116	RLCQ	5 months	ELISA	5 months	No correlation: cortisol, prolactin	Study additionally shows negative association between salivary cortisol and milk cortisol + prolactine (potential association between long-term stress)
[73]	2004	32	BDI + POMS	7–11 days	double enzyme fluorometric assay	7–11 days	↑ Cortisol (POMS-Anger)	
[75]	2005	119	TPSS, PSS	4–6 weeks	ELISA	4–6 weeks	↓ Prolactin, ↑ melatonin	SIgA was also correlated with milk prolactin, mutual relation between hormones were observed: lower milk prolactin was associated with higher milk melatonin
[71]	2024	26	EPDS, STAI	5 weeks	chemiluminescent immunoassay	1 and 5 weeks	↓ Cortisol (only week 1)	
[76]	2024	90	EPDS, ELSQ	5 and 12 months	ELISA	5 months	No correlation: cortisol	
[77]	2024	17 preterm 25 term	PSS, STAI, EPDS	6 months and at specific point ***	RP-UPLC MS/MS	6 months and at specific point ***	↑ Pregnenolone	
							No correlation: cortisol	
[78]	2023	73	CTQ, STAI, EPDS	3 trimester, 2, 6, 12 weeks	LC-MS/MS	2, 6, 12 weeks	↑ Cortisol	week 2—state anxiety week 6—adverse childhood experiences
[79]	2001	42	STAI	4 days	125-I RIA	4 days	↓ β-endorphin (state anxiety only)	No correlation for trait anxiety
[80]	2023	30 preterm 38 term 22 post-term	PSS	7 days	ELISA	7 days	↑ Cortisol	
[81]	22	38	PSS, EPDS	1, 3, 6 months	ELISA	1, 3, 6 months	No correlation: cortisol	
[72]	2024	55	STAI	1–8 months ^	ELISA	1–8 months ^	No correlation: oxytocin	
Ions (n = 5)								
[71]	2024	26	EPDS, STAI	5 weeks	ion selective electrodes (Na^+^ and K^+^)	1 and 5 weeks	No correlation: Na:K ratio	
[82]	2021	40	EPDS	7 days	ICP-MS	7 days	No correlation: Ca, Na, Fe, Se	
[83]	2008	64	STAI, EPDS	10 days	Hiatchi Modular Analytics ISE—ion selective method	10 days	↑ Na (only state anxiety)	
[84]	2017	150	STAI, EPDS	8–15 days	ion selective electrode	8–15 days	↑ Na (only state anxiety), Na:K ratio	
[85]	2008	163	EPDS	6 weeks	SPSS	2–12 weeks	↑ mammary gland permeability (Na:K ratio)	
RNA (n = 1)								
[86]	2021	80	NLEs, LSCR	27 ± 8 weeks of gestation	exoEasy Maxi Kit	6.1 ± 5.9 weeks	↑ negative events during pregnancy = ↓ microRNA	

**Abbreviations: AA**—arachidonic acid; **AAs**—amino acids; **ACT1**—actinobacteria such as *Bifidobacterium*, *Collinsella*, *Eggerthella*; **BDI**—Beck Depression Inventory; **BSSS**—Berlin Social Support Scale; **CES-D**—Center for Epidemiologic Studies Depression Scale; **CTQ**—Childhood Trauma Questionnaire; **DHA**—docosahexaenoic acid; **EGF**—epidermal growth factor; **ELSQ**—Early Life Stress Questionnaire; **EPA**—eicosapentaenoic acid; **EPDS**—Edinburgh Postpartum Depression Scale; **FA**—fatty acid; **GC**—gas chromatography; **GROα**—chemokine (C-X-C motif) ligand 1; **HPLC**—high-performance liquid chromatography; **HM**—human milk; **ICP**—ion-coupled plasma; **IgG**—immunoglobulin G; **IgM**—immunoglobulin M; **IL**—interleukin; **IR**—infra-red; **JTV**—Jeugd Trauma Vragenlijst; **LAB**—lactic acid bacteria such as *Lactobacillus*, *Lactococcus*, *Aerococcus*; **LC**—liquid chromatography; **LSCR**—Life Stressor Checklist—Revised; **MCP1**—monocyte chemotactic protein-1; **MID-IR**—mid infra-red transmission spectroscopy; **MIP1β**—Macrophage Inflammatory Protein-1-beta; **MS**—mass spectrometry; **PPD**—postpartum depression; **POMS**—Profile of Mood States; **PRAQ**—Pregnancy-Related Anxiety Questionnaire—Revised; **PRO1**—proteobacteria such as *Escherichia*, *Enterobacter*, *Serretia;* **PSS**—Perceived Stress Scale; **PUFA**—polyunsaturated fatty acid; **RID**—radial immunodiffusion; **RLCQ**—Recent Life Changes Questionnaire; **SIgA**—Secretory Immunoglobulin A; **STAI**—State-Trait Anxiety Inventory; **TGFβ2**—transforming growth factor beta two; **TNFα**—tumor necrosis factor alpha; **TPSS**—Tennessee Postpartum Stress Scale. * source is a cross-national analysis included due to presenting significant data; ** sample—infant stool; *** complete enteral nutrition (150 mL/kg/day) for preterm infants or recovery of birth weight for term infants; ^ estimated based on M and SD values reported in paper.

## Abbreviations

The following abbreviations are used in this manuscript:

AA	arachidonic acid
AAs	amino acids
ACT1	actinobacteria
BDI	Beck Depression Inventory
BSSS	Berlin Social Support Scale
CES-D	Center for Epidemiologic Studies Depression Scale
CTQ	Childhood Trauma Questionnaire
DHA	docosahexaenoic acid
EGF	epidermal growth factor
ELSQ	Early Life Stress Questionnaire
EPA	Eicosapentaenoic acid
EPDS	Edinburgh Postpartum Depression Scale
EVs	extracellular vesicles
FA	fatty acids
GC	gas chromatography
GROα	chemokine (C-X-C motif) ligand 1
HM	human milk
HMOs	human milk oligosaccharides
HPA	hypothalamic–pituitary–adrenocortical
HPLC	high-performance liquid chromatography
ICP	ion-coupled plasma
IgG	immunoglobulin G
IgM	immunoglobulin M
IL	interleukin
IR	infra-red
JTV	Jeugd Trauma Vragenlijst
LAB	lactic acid bacteria
LC	liquid chromatography
LSCR	Life Stressor Checklist—Revised
LSC-r	Life Stressor Checklist—Revised
MCP1	monocyte chemotactic protein-1
MID-IR	mid-infra-red transmission spectroscopy
MIP1β	Macrophage Inflammatory Protein-1-beta
MS	mass spectrometry
MUFA	monounsaturated fatty acid
POMS	Profile of Mood States
PPD	postpartum depression
PRAQ	Pregnancy-Related Anxiety Questionnaire—Revised
PRO1	proteobacteria
PSS	perceived Stress Scale
PUFA	polyunsaturated fatty acid
RID	radial immunodiffusion
RLCQ	Recent Life Changes Questionnaire
SIgA	secretory immunoglobulin A
SSRI	selective serotonin reuptake inhibitor
STAI	State-Trait Anxiety Inventory
TGFβ2	transforming growth factor beta two
TNFα	tumor necrosis factor alpha
TPSS	Tennessee Postpartum Stress Scale
